# Cardiovascular Diseases and Metabolic Medications in the Lebanese Population: A Post Hoc Analysis from a Nationwide Cross-Sectional Study

**DOI:** 10.3390/pharmacy12060171

**Published:** 2024-11-20

**Authors:** Rony M. Zeenny, Rachel Abdo, Chadia Haddad, Aline Hajj, Rouba Karen Zeidan, Pascale Salameh, Jean Ferrieres

**Affiliations:** 1 Department of Mathématiques Informatique et Télécommunications, Université Toulouse III, Paul Sabatier, INSERM, UMR 1295, F-31000 Toulouse, France; 2 INSPECT-LB (Institut National de Santé Publique, d’Épidémiologie Clinique Et de Toxicologie-Liban), Beirut 1103, Lebanon; rachelabdo@hotmail.com (R.A.); chadia_9@hotmail.com (C.H.); aline.hajj@hotmail.com (A.H.); roubakaren@gmail.com (R.K.Z.); pascalesalameh1@hotmail.com (P.S.); 3 Department of Pharmacy, American University of Beirut Medical Center, Beirut 1107, Lebanon; 4 Department of Psychomotricity, Faculty of Public Health, Lebanese University, Fanar 2611, Lebanon; 5 Gilbert and Rose-Marie Chagoury School of Medicine, Lebanese American University, Byblos 4504, Lebanon; 6 School of Health Sciences, Modern University of Business and Science, Beirut 7501, Lebanon; 7 Research Department, Psychiatric Hospital of the Cross, Jal El Dib 1525, Lebanon; 8 Faculté de Pharmacie, Université Laval, Québec, QC G1V 0A6, Canada; 9 Oncology Division, CHU de Québec-Université Laval Research Center, Québec, QC G1R 3S3, Canada; 10 Sharjah Institute of Medical Research, University of Sharjah, Sharjah 27272, United Arab Emirates; 11 Faculty of Pharmacy, Lebanese University, Hadat 1103, Lebanon; 12 Department of Primary Care and Population Health, University of Nicosia Medical School, Nicosia 2417, Cyprus; 13 Department of Cardiology and INSERM UMR 1295, Rangueil University Hospital, F-31059 Toulouse, France; jean.ferrieres@univ-tlse3.fr

**Keywords:** cardiovascular diseases, cross-sectional study, diabetes mellitus, hypoglycemic agents, hyperlipidemia, Lebanon, hypolipidemic agents, statins

## Abstract

Objective: This study assesses the association of metabolic drugs (specifically hypoglycemic and hypolipemic agents) with cardiovascular diseases (CVD) among the Lebanese population and patients’ subgroups. Methods: A nationwide cross-sectional retrospective study was carried out in Lebanon. The survey collected information on sociodemographic characteristics, lifestyles, comorbidities, and medication use. Logistic regression models were employed to analyze the data and determine associations between CVD and metabolic drugs. Stratification analyses were performed based on diabetes and dyslipidemia status. Results: The study found significant associations with CVD among the 2048 participants. Higher scores on the Lebanese Mediterranean Diet Score (LMDS; ORa = 1.06), hypertension (ORa = 1.71), diabetes (ORa = 1.75), dyslipidemia (ORa = 1.89), family history of CVD (ORa = 1.58), and smoking (previous: ORa = 1.63, current: ORa = 2.15) were linked to increased CVD odds. Higher income (intermediate: ORa = 0.64, high: ORa = 0.40) was inversely related to it. A subsequent model that included hypoglycemic and lipid-lowering medications yielded similar results. However, neither hypoglycemic nor lipid-lowering medications demonstrated a significant association with CVD risk. A third regression model was conducted by taking the classes of drugs as an independent variable. Also, the result revealed that all the classes of medication were not associated with the risk of CVD. Stratification by diabetes revealed LMDS and hypertension as risk factors in both groups. Among non-diabetic participants, dyslipidemia (ORa = 2.40), current smoking (ORa = 2.28), and higher income (intermediate: ORa = 0.57, high: ORa = 0.62) were linked to CVD. Among people with diabetes, a family history of CVD (ORa = 2.69) increased the CVD odds, while being an employer (ORa = 0.49) lowered it. Stratification by dyslipidemia showed consistent risk factors, and higher LMDS (ORa = 1.07), diabetes (ORa = 2.14), hypertension (ORa = 1.79), and previous smoking (ORa = 1.95) were linked to CVD without dyslipidemia. Being a female (ORa = 0.52) and having a lower income (ORa = 0.40) were associated with lower CVD odds in those with dyslipidemia. Subgroup analyses showed that medications were not significantly associated with CVD odds among patients with diabetes or hyperlipidemia. Conclusions: This study’s findings highlight the importance of addressing modifiable risk factors and socioeconomic factors to reduce the burden of CVD. Targeted interventions and longitudinal research are necessary to optimize preventive strategies and improve the management of CVD in individuals using hypoglycemic and hypolipemic agents in low- and medium-income countries.

## 1. Introduction

Cardiovascular diseases (CVD), including ischemic heart disease and stroke, remain the leading cause of global morbidity, mortality, and disability [1]. It is estimated that around 523 million people have CVD globally [2], with around 18.6 million deaths annually [2], accounting for 32% of all global deaths [3]. To put this into perspective, one person dies from CVD every 33 s [4].

Atherosclerosis stands as the predominant underlying condition in the majority of CVD cases [5]. Endothelial dysfunction, a pivotal factor in CVD development, is influenced by age, diabetes, dyslipidemia, hypertension, smoking, and sedentary lifestyle [6]. Oxidative stress and hyperglycemia-linked inflammation, notably exacerbated in individuals with diabetes mellitus (DM), worsen endothelial dysfunction, hastening the progression of atherosclerosis and escalating mortality rates among DM patients [7]. Patients with type 2 diabetes (T2D) face a two- to four-fold increased risk of developing CVD compared to healthy people [8]. DM affects over 463 million people worldwide [9] and is projected to reach 629 million people by 2045 [10]. Alarmingly, the overall risk for CVD mortality in patients with diabetes ranges from 1 to 3 in males and 2 to 5 in females, compared to those without DM [11].

Additionally, the literature consistently indicates a significant correlation between elevated plasma cholesterol levels and increased atherosclerotic disease risk [12]. Hypercholesterolemia, mainly due to oxidized low-density lipoprotein (LDL), impairs endothelial function, causing vasoconstriction and increasing platelet reactivity and hypercoagulability [13]. Consequently, dyslipidemia doubles the likelihood of developing CVD [14] and contributes to approximately 4 million CVD-related deaths worldwide [15].

The primary goal of CVD management lies in both prevention and early diagnosis. Although addressing behavioral risk factors, including tobacco use, unhealthy diet, obesity, physical inactivity, and excessive alcohol consumption, is crucial, it is not entirely sufficient [3].

Pharmacological strategies for managing T2DM aim to optimize glucose control, minimize hypoglycemia and adverse effects, and prevent CV events [16]. Treatment options range from traditional agents, such as insulin, metformin, sulfonylureas, and thiazolidinediones, to newer alternatives, such as glucagon-like peptide-1 receptor agonists (GLP-1 RA), sodium glucose cotransporter-2 inhibitors (SGLT2), and dipeptidylpeptidase-4 inhibitors (DPP4) [17].

Metformin is a first-line treatment for T2DM [18] and is linked to reduced mortality by lowering CVD risk [18]. A meta-analysis found that the metformin group had a slightly decreased incidence of fatal myocardial infarction (MI) compared to other hypoglycemic drugs [19]. Similarly, SGLT-2 inhibitors reduced all-cause and CV mortality [20]. GLP-1 agonists exhibit favorable effects on CVD, heart failure (HF), and kidney outcomes [17]. Thus, they are recommended for secondary prevention in T2DM patients with existing CVD [17]. Dipeptidylpeptidase-4 (DPP4) inhibitors generally have no significant effect on CV outcomes [21]. Finally, insulin remains widely used to treat T2DM in patients with CVD [17], with meta-analyses indicating no increased risk of CV mortality or MI associated with its use [22].

Similarly, lipid-lowering therapy (LLT) is a pivotal and highly effective pharmacological cornerstone in managing atherosclerotic cardiovascular diseases (ASCVD) [23]. Lowering LDL-C remains a key strategy in addressing dyslipidemia [15,24,25], with a 1 mmol/L reduction leading to a 20–25% decrease in ASCVD risk over the initial five-year period [26].

Agents such as statins, ezetimibe, and novel PCSK9 inhibitors have improved lipid profiles and reduced cardiovascular events and mortality [27,28]. Statins, in particular, are effective for both primary and secondary CVD prevention [27,29]. Long-term fibrate use achieves an approximate 22% reduction in the rate of non-fatal MI [30]. Unfortunately, adherence and persistence to LLT, particularly statins, remain suboptimal, negatively impacting clinical outcomes and residual CV risk [31]. Persistence rates are low at 23.3% in primary [32] and 36.8% in secondary prevention, with similarly low statin use among patients with diabetes [32]. Poor long-term statin persistence continues to be a challenge for patients at high risk of CVD, including those with elevated TG [33]. Financial constraints [34] and statin intolerance due to side effects also contribute to non-adherence [34]. Polypill treatments show better cost-effectiveness than statin-only regimens [35]. The relationship between diabetes, hyperlipidemia, and cardiovascular disease (CVD) is well established [36], but the specific impact of metabolic drugs used to manage these conditions on CVD risk remains unclear. While medications, such as metformin and SGLT2 inhibitors, have shown promise in reducing CVD risk [37], their precise effects, influenced by dosage, timing, and patient characteristics, are not fully understood. In Lebanon, the effects of metabolic drug use (hypoglycemic and hypolipemic agents) on CVD risk remain largely uninvestigated. While international research has addressed this [37,38], the Lebanese population’s unique treatment patterns and outcomes have not been explored. This study seeks to bridge the existing gap by investigating the potential association between the use of metabolic drugs (hypoglycemic and hypolipidemic agents) and CVD risk in Lebanese patients.

The central hypothesis is not that the population is improperly treated but rather to explore how these medications affect CVD risk and whether certain risk factors persist even among treated patients. Thus, this study aims to evaluate this association among the Lebanese population and specific patient subgroups, providing a clearer understanding of how these medications influence CVD risk in this context.

## 2. Methods

### 2.1. Design and Population

The methodology used in this study is described elsewhere [39,40,41]. Data were gathered via a cross-sectional survey conducted from September 2013 to October 2014, utilizing a multistage cluster sampling method throughout Lebanon [39]. The list of 2789 circumscriptions (villages or communities considered clusters) across Lebanon was received from the Lebanese Central Administration of Statistics [42]. Using an automatic random number generator, 100 circumscriptions were chosen at random from the complete list of 2789 across Lebanon. Within each selected circumscription, local authorities provided a list of the residents. Accordingly, a software application randomly chose individuals aged 18 or older. Authorities from each community or village were contacted, and selected inhabitants from a provided list were invited to meet with the researchers in a designated community space, such as a church or mosque. Participants who declined to participate were substituted with others from the list. Upon obtaining oral and written consent, participants completed face-to-face interviews. Individuals known to be suffering from mental disorders or learning disabilities were excluded. The institutional review board of the Lebanese University waived the need for ethical approval, as this was an observational study with no identifiable data collection.

### 2.2. Sample Size

The sample size was initially calculated to measure the prevalence of cardiovascular diseases and their risk factors using Epi Info™ (Version 7, Centers for Disease Control and Prevention, Atlanta, GA, USA) [43]. A minimal sample size of n = 1200 was required to estimate the prevalence of cardiovascular diseases in Lebanon in an adult population aged 40 years and more. The prevalence of arterial hypertension of 41.3% among individuals aged 50 years and older was used as a baseline [44]. We accounted for an allowable margin of ±4% variation from the aforementioned prevalence, along with a 95% confidence interval (CI), while factoring in the two-stage sampling approach.

### 2.3. Data Collection

Self-reported data were gathered through a standardized questionnaire concerning sociodemographic characteristics. It included the following: sociodemographic factors (e.g., age, gender, marital status, educational level, region of dwelling, occupation, and socioeconomic background), history of heart diseases, underlying medical history, and use of hypoglycemic and hypolipemic medications before the CVD diagnosis. Additional information, such as social habits (e.g., smoking and physical activities), was also collected.

Socioeconomic status was categorized into four levels based on total household monthly income, as follows: low (<USD 400, equivalent to the minimum wage in Lebanon), intermediate low (USD 400–1000), intermediate high (USD 1000–2000), and high (>USD 2000).

Psychological distress was also assessed using the Beirut Distress Scale (BDS-22), a tool specifically developed and validated for the Lebanese population. The BDS-22 included 22 items with a 4-point Likert response scale (0–3). Scores on the BDS-22 range from 0, indicating no psychological distress, to 66, representing the highest level of distress [45].

The study also utilized the Lebanese Mediterranean Diet Score (LMDS), a 16-item questionnaire designed to measure adherence to a Mediterranean diet within the context of Lebanese food consumption. The scale encompasses all major dietary groups, including the five basic food groups most Lebanese people eat. Higher scores suggest maximum adherence to the MedDiet, with scores ranging from 0 to 52 [46]. Since alcohol consumption is believed to be prohibited by religion, the study did not measure any alcohol intake because it is likely that alcohol consumption would go unreported.

### 2.4. Definitions

#### Cardiovascular Disease Definition

Participants with a history of MI, percutaneous coronary intervention, or coronary artery bypass graft were deemed eligible for the study. Angina was identified according to the “definite angina” criteria set by the Rosa Angina Questionnaire [47]. The occurrence of MI, angina pectoris, percutaneous coronary intervention, or coronary artery bypass graft was aggregated to determine the lifetime prevalence of the coronary disease. In addition, cerebrovascular diseases were identified based on a positive response to the question, “Has the doctor ever told you that you had a stroke or a ministroke?” The main dependent variable was the presence of CVD, covering both coronary and cerebrovascular diseases.

### 2.5. Definitions of Risk Factors for CVD

Hypertension: Hypertension was identified by either a self-reported history of the condition or multiple readings with systolic blood pressure of ≥140 mmHg or diastolic blood pressure of ≥90 mmHg [48].

Diabetes: Individuals received random capillary blood glucose (RCBG) testing using Accu-Check^®^ Performa (Roche Diagnostics GmbH, Mannheim, Germany). Diabetes was identified by RCBG levels exceeding 200 mg/dL or self-reported use of glucose-lowering medications [49].

Dyslipidemia: Dyslipidemia was identified through either a self-reported history of hyperlipidemia, regardless of current LLT, or recent blood test results showing elevated lipid levels. Hypercholesterolemia was considered when having an LDL-C of 100 mg/dL or more [50]. In contrast, hypertriglyceridemia was considered when having a triglyceride value of 200 mg/dL or more [50].

Smoking: Current smokers were described as individuals who had smoked tobacco within the previous 12 months, including recent quitters. Former smokers referred to those who have been smoke-free for over a year.

History of CVD: A history of heart disease was defined as any self-reported occurrence of MI, stenting, angioplasty, or coronary artery bypass graft surgery. A family history of early-onset CVD was considered if a first-degree relative had experienced any cardiovascular event before the age of 55 for men or 65 for women [51].

Physical activity: Individuals were considered physically active if they regularly engaged in moderate-intensity physical activity for at least 150 min or vigorous-intensity physical activity for 75 min weekly [52].

### 2.6. Anthropometric Measurements

Trained medical students conducted the measurements, which included anthropometric measurements, such as weight (in kg), height (in m), and waist circumference (WC; in cm). Body mass index (BMI) was calculated by dividing weight in kilograms by height in square meters. BMI was classified into three categories: normal weight (BMI < 25 kg/m^2^), overweight (25 kg/m^2^ ≤ BMI < 30 kg/m^2^), and obese (BMI ≥ 30 kg/m^2^). WC thresholds for defining abdominal obesity were 93.5 cm for men and 92.5 cm for women [53]. Blood pressure (systolic and diastolic) was measured twice with a validated Omron^®^ M6 Comfort (Omron^®^, Kyoto, Japan) using a standardized guideline [54].

### 2.7. Statistical Analysis

Prior to statistical analysis, two independent observers conducted a double-check to ensure the quality of the questionnaire data, and an additional audit was carried out on a randomly selected 5% sample of the questionnaires. Population data from the Lebanese Ministry of Social Affairs and the Central Administration of Statistics provided theoretical percentages for the distribution of adults across Lebanese regions, taking into account gender, sex, age, and residential region [42]. Individual participants were then weighted to align with this distribution, ensuring that the sample reflected the demographic characteristics of the Lebanese population. The weighting for the underrepresented participants in the sample was increased, while it was reduced for those overrepresented, maintaining the overall sample unchanged. Data analysis was conducted using SPSS software version 25. Descriptive statistics included counts and percentages for categorical variables, while means and standard deviations were calculated for continuous variables. Chi-square and Fisher’s exact tests were employed to compare categorical variables, while the Student’s *t*-test was applied to compare continuous variables between two groups. A *p*-value < 0.05 was considered statistically significant. Furthermore, Bonferroni adjustments were applied to account for multiple comparisons, minimizing the risk of Type I errors.

Three logistic regression analyses were performed using the Enter method, with CVD set as the dependent variable. The first model included the sociodemographic variables, LMDS, and smoking status as independent variables. The second model expanded on this by adding the use of hypoglycemic and lipid-lowering medications. In the third model, the classes of hypoglycemic and lipid-lowering medications were included as independent variables. Variables with a *p* < 0.1 in the bivariate analysis were selected as essential variables to include in the model, aiming to reduce potential residual confounding. In addition, two stratification analyses with diabetes and dyslipidemia were performed, taking the CVD as the dependent variable. Before conducting the regression, we ensured the absence of collinearity between the covariates to prevent over-adjustment.

## 3. Results

### 3.1. Sociodemographic Characteristics of the Sample Population

The analysis assessed sociodemographic characteristics across a range of variables, contrasting participants with cardiovascular diseases (CVD) against those without. The sample consisted of 2048 participants, with 21.5% having CVD. The study found significant associations between cardiovascular diseases (CVD) and various sociodemographic factors. The prevalence of CVD was higher among males (53.4%) than females (46.6%; *p* = 0.021), and older individuals, mainly those aged 60+, had a higher rate of CVD (37%) than younger age groups (*p* < 0.001). Married individuals had a higher likelihood of CVD (23.5%) compared to single, divorced, or widowed (19.3%; *p* = 0.023). Participants with lower education levels and lower incomes showed higher rates of CVD, with 30.3% in the complementary education group (*p* < 0.001) and 33.2% in the low-income group (*p* = 0.001). Geographically, CVD prevalence was higher in Bekaa (28.6%) and South Lebanon (29.1%; *p* < 0.001), and urban residents had more CVD cases (25.4%) than rural residents (18.6%; *p* = 0.003). Additionally, unemployed and retired participants were more likely to have CVD (24.5%) compared to employed individuals (*p* = 0.011).

### 3.2. Risks Factors

Risk factors associated with CVD are summarized in Table 1. Participants with CVD had higher rates of family history of CVD (28.4% vs. 17.5%, *p* < 0.001), hypertension (36.7% vs. 14.7%, *p* < 0.001), diabetes (37.2% vs. 17.9%, *p* < 0.001), and dyslipidemia (40.6% vs. 17.0%, *p* < 0.001) compared to those without CVD. Moreover, a significantly greater proportion of smokers (22.9% vs. 17.0%, *p* < 0.001) had CVD compared to non-smokers. Individuals on hypoglycemic medications (43.3% vs. 19.8%, *p* < 0.001) and lipid-lowering medications (44.9% vs. 19.4%, *p* < 0.001) had a higher likelihood of CVD compared to those not using these medications. Specifically, patients taking sulfonylurea (45.5% vs. 20.9%, *p* < 0.001), biguanides (metformin; 43.3% vs. 20.2%, *p* < 0.001), insulin (53.3% vs. 21.3%, *p* = 0.007), statins (46.4% vs. 19.6%, *p* < 0.001), and fibrates (39.1% vs. 21.4%, *p* = 0.046) had a significantly higher rate of CVD than those not taking these medications. Finally, the mean values for BMI (27.52 vs. 26.66, *p* < 0.001), LMDS score (32.11 vs. 30.59, *p* < 0.001), and BDS-22 score (37.74 vs. 31.24, *p* < 0.001) were all significantly higher in participants with CVD than in those without.

### 3.3. Multivariable Analysis

A first logistic regression, taking CVD as the dependent variable and sociodemographic and LMDS variables as independent variables, indicated that higher LMDS scores (ORa = 1.06), hypertension (ORa = 1.71), diabetes (ORa = 1.75), dyslipidemia (ORa = 1.89), a family history of CVD (ORa = 1.58), and being a former or current smoker (ORa = 1.63 and ORa = 2.15, respectively) were significantly associated with increased odds of CVD. Conversely, lower intermediate (ORa = 0.64) and high (ORa = 0.40) monthly incomes were significantly linked to reduced odds of developing CVD (Table 2, Model 1).

A second logistic regression, taking CVD as the dependent variable and the use of hypoglycemic and lipid-lowering medications as independent variables, showed that higher LMDS (ORa = 1.06), having hypertension (ORa = 1.69), diabetes (ORa = 1.69), dyslipidemia (ORa = 1.80), a family history of CVD (ORa = 1.58), and being a former or current smoker (ORa = 1.64 and ORa = 2.15, respectively) were significantly associated with higher CVD odds. However, lower intermediate (ORa = 0.64) and high (ORa = 0.40) monthly income were associated with lower CVD odds. Both hypoglycemic and lipid-lowering medications did not show any significant association with CVD risk (Table 2, Model 2).

A third logistic regression, taking CVD as the dependent variable and the classes of hypoglycemic medications and lipid-lowering medications as independent variables, showed similar results to the previous model. However, in this model, the drug classes did not show a statistically significant relationship with CVD risk (*p* > 0.05 for all; Table 2, Model 3).

### 3.4. Stratified Analysis

When stratifying the analysis by diabetes status (presence or absence of diabetes), the results revealed significant associations between higher LMDS scores and hypertension with increased odds of CVD in both groups. Among participants without diabetes, having dyslipidemia (ORa = 2.40) and being a current (ORa = 2.28) or a previous smoker (ORa = 1.28) were associated with increased CVD odds. However, lower intermediate (ORa = 0.57), higher intermediate (ORa = 0.62), or high monthly income (ORa = 0.36) were all linked to reduced CVD odds. Among participants with diabetes, a family history of CVD (ORa = 2.69) increased CVD odds, whereas being employed (ORa = 0.49) reduced the odds. Notably, among diabetic patients, lipid-lowering medications showed no significant association with CVD risk (Table 3).

The stratification analysis by dyslipidemia status (presence or absence of dyslipidemia) revealed significant associations between hypertension, family history of CVD, and smoking, with higher CVD odds in both groups. Additionally, increased monthly income was significantly correlated with lower CVD odds. Among participants without dyslipidemia, a higher LMDS score (ORa = 1.07), the presence of diabetes (ORa = 2.14), hypertension (ORa = 1.79), and previous smoking status (ORa = 1.95) were significantly associated with increased CVD odds. Among participants with dyslipidemia, female gender (ORa = 0.52) and a lower intermediate income (ORa = 0.40) or high income (ORa = 0.35) were significantly linked to reduced CVD odds. Notably, among patients with hyperlipidemia, hypoglycemic agents were not significantly linked to CVD risk (Table 4).

## 4. Discussion

This study is the first population-based research in Lebanon and the Middle East to evaluate the association between CVD and metabolic drugs, including hypoglycemic and hypolipemic agents. This study assessed the risk factors associated with CVD in the Lebanese population. The multivariable logistic regression analysis, with hypoglycemic medications use as the dependent variable, showed that age and hypertension were its main determinants. In contrast, the analysis for lipid-lowering medication use showed that age, hypertension, and hyperlipidemia were its main determinants. These findings align with existing literature regarding the profile of patients treated with metabolic medications [55].

Our study identified several factors significantly associated with higher odds of CVD, including male gender, older age, being married, urban residence, particularly in Beirut, unemployment or retirement, lower education levels, and lower monthly income. These findings are consistent with several studies and are probably pre-existent among participants, constant over time, and difficult or impossible to change. CVD rates vary between genders and age groups [56]. Age plays a crucial role in cardiovascular health deterioration, leading to an elevated risk of CVD in older individuals [56]. Moreover, our findings align with those of Dhindsa et al. [57]. This supports the association between marriage and a lower risk of CVD; however, the underlying mechanisms remain unclear. Urbanization is also a significant predictor of CVD mortality rates [58]. Urban areas have higher pollution levels than rural areas [58]. Numerous epidemiological studies on air pollution and CVD from both developed and developing countries [59], including Lebanon [60], have demonstrated a potential link between outdoor air pollution and CVD [60].

Our results also align with previous research [1], showing that low-income and low socioeconomic status (SES) significantly increase CVD risk, incidence, and mortality rates among both genders. A significant reason is that patients with low SES often face barriers to accessing healthcare services [1], resulting in delays or inadequate CVD care and treatment. Additionally, low SES is also associated with a higher likelihood of CV risk factors, including smoking, alcohol consumption, hypertension, physical inactivity, obesity, diabetes, an unhealthy diet, and depression [1]. Similarly, our findings align with more literature demonstrating that higher distress has been linked to mortality and CVD [61].

We also identified a strong correlation between CVD and a family history of CVD, hypertension, diabetes, and dyslipidemia. Many risk factors have been linked to the onset of CVD, such as hypertension, diabetes [62], and dyslipidemia [62,63].

Additionally, participants taking hypoglycemic medications (particularly sulfonylurea, biguanide, and insulin) and those taking lipid-lowering medications (particularly statins and fibrates) more often reported having CVD than participants who did not take them. This could be explained by the fact that participants on metabolic drug medications have a higher CVD risk profile and probability of using these medications compared to those not on current medications (who probably do not need them), and this confounding by indication is widely known in the real-world effect of medications [64]. The remaining residual risk is also an explanation [65], likely due to inadequate treatment or adherence to prescribed medications [66] or other risk factors [67].

However, when we added the hypoglycemic medications and the lipid-lowering medications to the second model and the significant classes of hypoglycemic and lipid-lowering medications to the third model in the multivariate logistic regression, we obtained the same remaining variables, with no significant differences between the hypoglycemic and lipid-lowering medications, nor between their respective medications’ classes. This may be due to the low percentage in each class category. Furthermore, this result could mean that the intake of these medications is equalizing CVD odds in the Lebanese population despite the variability of risk factors [68]. Moreover, subgroup analyses indicated that hypoglycemic or hypolipemic agents were not significantly associated with decreased CVD odds among participants, as expected from a treatment. This was true for subgroups with or without diabetes and with or without hyperlipidemia, respectively. These findings raise questions about correct pharmacological management, medication adherence [69], and other concomitant secondary prevention measures [70]. We could not identify previous research that studied these factors using similar methods, and further studies are necessary to confirm their importance. Future prospective research is suggested to verify and further explain these findings, as cross-sectional studies cannot display these complex chronological phenomena [71].

Other than the previously reported sociodemographic factors, BMI, diet (higher LMDS scores), and smoking status (smokers and ex-smokers) were also associated with increased odds of CVD. This is consistent with previous studies showing that obesity significantly predicts CVD mortality [72]. Moreover, according to the CDC, smoking is a significant contributor to CVD, accounting for approximately one of every four deaths from CVD [73]. Nevertheless, smokers who quit begin to improve their heart health and reduce their risk for CVD [73]. A possible explanation for why ex-smoker participants had higher CVD in our study, as opposed to other studies, is that they may have recently stopped smoking because of higher CVD, and due to the cross-sectional study type, we were unable to capture the long-term benefits of quitting smoking in reducing CVD. An unhealthy diet is a major risk factor for CVDs [74]; however, individuals diagnosed with a serious illness may adopt healthier lifestyles, such as improving their diet and quitting smoking [75]. This phenomenon, known as reverse causality in cross-sectional studies, deserves to be assessed in further prospective cohort studies of the Lebanese population.

Finally, the profile of a diabetic participant with CVD is being unemployed, having a family history of CVD, hypertension as comorbidity, current healthy nutritional behavior, and being a smoker. Compared with people without diabetes, patients with diabetes have twice the risk of CVD, which tends to occur earlier and with more severe effects [8]. Compared to individuals without T2DM, those with T2DM have a two-fold increased risk of mortality from heart disease and stroke [76]. Moreover, smoking and diabetes have a significant interactive effect, amplifying the risk of subsequent CVD events [77]. This study found that diabetes and hypertension raise the risk of CVD. Previous studies [78] have highlighted a synergistic additive effect of these conditions on coronary heart disease. Therefore, a combined intervention of diabetes and hypertension management and smoking cessation mainly impacts CVD outcomes [79]. Similarly, the profile of a dyslipidemia participant with CVD is having a family history of CVD, hypertension as comorbidity, and being a smoker [80].

### Limitations and Strengths

The results of this study should be interpreted in light of the following limitations and strengths. Firstly, our post hoc study generated hypotheses that warrant further investigation in prospective studies. Similar to all cross-sectional studies, demonstrating temporality and causality is challenging. However, temporal relationships are plausible for associations such as family history, age, gender, and education level. Another limitation is the possibility of selection bias during sampling, despite our efforts to adopt random selection. This bias may be particularly relevant for certain missing values, especially from participants who opted not to respond to questions concerning socioeconomic status.

Additionally, the population-based design of this study limited the ability to measure the prevalence of asymptomatic CVD. Since CVD data and most risk factors were self-reported, misclassification bias is possible. However, we tried to collect information from all available diagnostic and laboratory tests.

Furthermore, the study did not account for lifestyle changes before the survey or the duration of medication intake, which may introduce bias regarding the participant’s health status and treatment history at the time of the study [81]. In addition, certain variables, such as dyslipidemia, were not stratified into stages, which may limit the granularity of the findings. While we performed multivariable analyses to account for confounding effects, residual confounding remains possible. Finally, our sample size is suitable for bivariate and multivariate analyses; however, it may need more power for some multivariable analyses considering medication classes. Therefore, prospective studies that address these limitations are necessary to validate our results.

Despite these limitations, our study offers several strengths. Its conclusions benefit from the generalizability provided by the sample size and representativeness of the study population. Moreover, the questionnaire covered multiple dimensions, enabling us to examine the relationship between CVD and various sociodemographic, behavioral, and health-related risk factors and generate hypotheses to be further researched.

## 5. Conclusions

Our study revealed significant associations between CVD and lifestyle habits, comorbidities, and socioeconomic status. Key risk factors included higher LMDS scores, hypertension, diabetes, dyslipidemia, smoking, and a CVD family history, while higher income was associated with a protective effect. Although specific hypoglycemic and lipid-lowering medications were not independently associated with CVD, taking lipid-lowering medications was linked to an increased CVD risk. The stratification analyses by diabetes and dyslipidemia reinforced the findings. They emphasized the need to address modifiable factors, such as lifestyle hypertension, dyslipidemia, and diabetes, to reduce the burden of CVD. Further research is needed to optimize prevention and treatment strategies for patients with CVD.

## Figures and Tables

**Table 1:** Risk factors for participants with and without cardiovascular disease (CVD).

	No CVD *n* = 1608 (78.5%)	CVD *n* = 440 (21.5%)	Total N 2048 (100%)	*p*-Value
**Family History of CVD**				**<0.001**
Yes	538 (71.6%)	213 (28.4%)	751 (36.7%)	
No	1070 (82.4%)	227 (17.5%)	1297 (63.3%)	
**Hypertension**				**<0.001**
Yes	400 (63.3%)	232 (36.7%)	632 (30.9%)	
No	1208 (85.3%)	208 (14.7%)	1416 (69.1%)	
**Diabetes Mellitus**				**<0.001**
Yes	236 (62.8%)	140 (37.2%)	376 (18.4%)	
No	1372 (82.1%)	300 (17.9%)	1672 (81.6%)	
**Dyslipidemia**				**<0.001**
Yes	230 (59.4%)	157 (40.6%)	387 (18.9%)	
No	1378 (83.0%)	283 (17.0%)	1661 (81.1%)	
**Overall Smoking**				**<0.001**
Yes	776 (77.1%)	230 (22.9%)	1006 (49.1%)	
Previous	105 (62.9%)	62 (37.1%)	167 (8.2%)	
No	727 (83.0%)	148 (17.0%)	875 (42.7%)	
**Regular Physical Activity**				0.708
Yes	522 (78.0%)	147 (22.0%)	669 (32.7%)	
No	1086 (78.8%)	293 (21.2%)	1379 (67.3%)	
**Hypoglycemic medications**				**<0.001**
Yes	89 (56.7%)	68 (43.3%)	157 (7.7%)	
No	1519 (80.3%)	372 (19.7%)	1891 (92.3%)	
**Type of hypoglycemic medications**				
**Sulfonylurea**	**30 (54.5)**	**25 (45.5)**	**55 (2.7%)**	**<0.001**
**Biguanides (metformin)**	**68 (56.7)**	**52 (43.3)**	**120 (5.9%)**	**<0.001**
Thiazolidinedione	1 (50)	1 (50)	2 (0.1%)	0.385
Meglitinides	2 (100)	0	2 (0.1%)	1
DPP4 inhibitors	16 (72.7)	6 (27.3)	22 (1.1%)	0.601
**Insulin**	**7 (46.7)**	**8 (53.3)**	**15 (0.7%)**	**0.007**
**Lipid-lowering medications**				**<0.001**
Yes	97 (55.1%)	79 (44.9%)	176 (8.6%)	
No	1511 (80.7%)	361 (19.3%)	1872 (91.4%)	
**Type of lipid-lowering medications**				
**Statins**	**82 (53.6)**	**71 (46.4)**	**153 (7.5%)**	**<0.001**
**Fibrates**	**14 (60.9)**	**9 (39.1)**	**23 (1.1%)**	**0.046**
Cholesterol absorption inhibitor	1 (100)	0	1 (0%)	1
Omega 3	6 (85.7)	1 (14.3)	7 (0.3%)	0.709
	Mean ± SD	Mean ± SD	Mean ± SD	
**BMI (kg/m^2^)**	**26.66 ± 4.88**	**27.52 ± 5.36**	**26.85 ± 4.99**	**0.001**
**LMDS**	**30.59 ± 4.48**	**32.11 ± 4.72**	**30.98 ± 4.59**	**<0.001**
**BDS-22**	**31.24 ± 10.08**	**37.74 ± 13.15**	**32.60 ± 11.11**	**<0.001**

Abbreviations: BDS-22: Beirut Distress Scale 22; BMI: body mass index; CVD: cardiovascular disease; DPP4: dipeptidylpeptidase-4 inhibitors; LMDS: Lebanese Mediterranean Diet Score; SD: standard deviation. ***p*-values in bold:** Statistically significant values.

**Table 2:** Multivariable analysis—Models 1, 2, and 3.

**Model 1: Logistic regression taking the CVD as the dependent variable with the sociodemographic, LMDS, and smoking status variables as independent variables.**		
	**CVD as the Dependent Variable**	
	**ORa (95% CI)**	***p*-Value**
Gender (female vs. male *)	0.84 (0.63–1.12)	0.239
Marital status (married vs. single *)	0.82 (0.61–1.10)	0.185
Education level (university vs. school *)	1.09 (0.79–1.51)	0.581
Work status (employed vs. unemployed *)	0.78 (0.58–1.07)	0.130
Age	0.99 (0.98–1.00)	0.610
**LMDS**	1.06 (1.03–1.09)	**<0.001**
BMI	1.00 (0.98–1.03)	0.505
Hypertension (yes vs. no *)	1.71 (1.25–2.33)	**0.001**
Diabetes (yes vs. no *)	1.75 (1.26–2.42)	**0.001**
Dyslipidemia (yes vs. no *)	1.89 (1.37–2.59)	**<0.001**
Family history of CVD (yes vs. no *)	1.58 (1.21–2.07)	**0.001**
Smoking status (previous vs. no *)	1.63 (1.21–2.20)	**0.001**
Smoking status (yes vs. no *)	2.15 (1.36–3.41)	**0.001**
Monthly income (lower intermediate vs. low *)	0.64 (0.45–0.91)	**0.013**
Monthly income (higher intermediate vs. low *)	0.85 (0.60–1.20)	0.370
Monthly income (high vs. low *)	0.40 (0.25–0.62)	**<0.001**
Variables entered in the model: age, BMI, diabetes, dyslipidemia, education level, family history of CVD, gender, hypertension, income, LMDS, marital status, smoking status, and work status		
**Model 2: Logistic regression taking the CVD as the dependent variable and the sociodemographic variables, LMDS, smoking status, and hypoglycemic medications and lipid-lowering medications as independent variables**		
	**CVD as the Dependent Variable**	
	**ORa (95% CI)**	***p*-Value**
Gender (female vs. male *)	0.85 (0.63–1.13)	0.255
Marital status (married vs. single *)	0.82 (0.61–1.10)	0.195
Education level (university vs. school *)	1.08 (0.78–1.50)	0.611
Work status (employed vs. unemployed *)	0.79 (0.58–1.08)	0.142
Age	0.99 (0.98–1.00)	0.572
**LMDS**	**1.06 (1.03–1.09)**	**<0.001**
BMI	1.01 (0.98–1.04)	0.491
Hypertension (yes vs. no *)	**1.69 (1.24–2.32)**	**0.001**
Diabetes (yes vs. no *)	**1.69 (1.14–2.50)**	**0.009**
Dyslipidemia (yes vs. no *)	**1.80 (1.24–2.62)**	**0.002**
Family history of CVD (yes vs. no *)	**1.58 (1.21–2.06)**	**0.001**
Smoking status (previous vs. no *)	**1.64 (1.22–2.21)**	**0.001**
Smoking status (yes vs. no *)	**2.15 (1.36–3.41)**	**0.001**
Monthly income (lower intermediate vs. low *)	**0.64 (0.45–0.91)**	**0.013**
Monthly income (higher intermediate vs. low *)	0.85 (0.60–1.21)	0.363
Monthly income (high vs. low *)	**0.40 (0.26–0.62)**	**<0.001**
Hypoglycemic medications (yes vs. no *)	1.11 (0.68–1.81)	0.656
Lipid-lowering medications (yes vs. no *)	1.08 (0.64–1.82)	0.774
Variables entered in the model: age, BMI, diabetes, dyslipidemia, education level, family history of CVD, gender, hypertension, hypoglycemic medications, income, lipid-lowering medications, LMDS, marital status, smoking status, and work status.		
**Model 3: Logistic regression taking the CVD as the dependent variable and the sociodemographic variables, LMDS, smoking status, and the classes of medications as independent variables**		
	**CVD as the Dependent Variable**	
	**ORa (95% CI)**	***p*-Value**
Gender (female vs. male *)	0.85 (0.64–1.14)	0.288
Marital status (married vs. single *)	0.80 (0.60–1.08)	0.162
Education level (university vs. school *)	1.06 (0.77–1.47)	0.709
Work status (employed vs. unemployed *)	0.81 (0.59–1.10)	0.182
Age	0.99 (0.98–1.00)	0.529
**LMDS**	**1.06 (1.03–1.09)**	**<0.001**
BMI	1.01 (0.98–1.03)	0.464
Hypertension (yes vs. no *)	**1.68 (1.23–2.30)**	**0.001**
Diabetes (yes vs. no *)	**1.68 (1.15–2.46)**	**0.007**
Dyslipidemia (yes vs. no *)	**1.74 (1.19–2.53)**	**0.004**
Family history of CVD (yes vs. no *)	**1.59 (1.22–2.08)**	**0.001**
Smoking status (previous vs. no *)	**1.65 (1.22–2.22)**	**<0.001**
Smoking status (yes vs. no *)	**2.20 (1.38–3.49)**	**0.001**
Monthly income (lower intermediate vs. low *)	**0.65 (0.45–0.92)**	**0.001**
Monthly income (higher intermediate vs. low *)	**0.85 (0.60–1.21)**	**<0.001**
Monthly income (high vs. low *)	**0.40 (0.25–0.62)**	**0.014**
Sulfonylurea (yes vs. no *)	0.70 (0.34–1.46)	0.345
Biguanide (metformin) (yes vs. no *)	1.38 (0.78–2.44)	0.267
Insulin (yes vs. no *)	1.06 (0.29–3.75)	0.928
Statins (yes vs. no *)	1.32 (0.80–2.18)	0.269
Fibrates (yes vs. no *)	0.61 (0.21–1.80)	0.375
The model entered the following variables: age, biguanide (metformin), BMI, diabetes, dyslipidemia, education level, family history of CVD, fibrates, gender, hypertension, income, insulin, LMDS, marital status, smoking status, statins, sulfonylurea, and work status.		

* Reference group. Abbreviations: BMI: body mass index; CI: confidence interval; CVD: cardiovascular disease; LMDS: Lebanese Mediterranean Diet Score; ORa: adjusted odds ratio. ***p*-values in bold:** Statistically significant values.

**Table 3:** Multivariable analysis stratified by having diabetes.

Logistic Regression Taking CVD as the Dependent Variable				
	Not Having Diabetes		Having Diabetes	
	ORa (95% CI)	*p*-Value	ORa (95% CI)	*p*-Value
Gender (female vs. male *)	1.03 (0.74–1.45)	0.823	0.55 (0.29–1.03)	0.063
Marital status (married vs. single *)	0.78 (0.55–1.10)	0.159	1.16 (0.56–2.39)	0.677
Education level (university level vs. school level *)	1.06 (0.73–1.54)	0.742	1.08 (0.51–2.32)	0.827
Work status (employed vs. unemployed *)	0.91 (0.64–1.30)	0.628	**0.49 (0.24–0.97)**	**0.043**
Age	0.99 (0.98–1.01)	0.686	0.99 (0.97–1.01)	0.477
LMDS	**1.05 (1.01–1.09)**	**0.003**	**1.08 (1.01–1.14)**	**0.010**
BMI	1.02 (0.98–1.05)	0.217	0.97 (0.92–1.03)	0.410
Hypertension (yes vs. no *)	**1.77 (1.22–2.57)**	**0.002**	**1.90 (1.01–3.58)**	**0.046**
Dyslipidemia (yes vs. no *)	**2.40 (1.53–3.77)**	**<0.001**	1.06 (0.52–2.17)	0.854
Family history of CVD (yes vs. no *)	1.29 (0.94–1.77)	0.103	**2.69 (1.54–4.70)**	**<0.001**
Smoking status (previous vs. no *)	**1.81 (1.28–2.56)**	**0.001**	1.14 (0.59–2.20)	0.682
Smoking status (yes vs. no *)	**2.28 (1.31–3.97)**	**0.003**	2.27 (0.91–5.64)	0.076
Lipid-lowering medications (yes vs. no *)	0.75 (0.39–1.46)	0.409	1.76 (0.81–3.82)	0.152
Monthly income (lower intermediate vs. low *)	**0.57 (0.38–0.85)**	**0.006**	0.89 (0.44–1.81)	0.768
Monthly income (higher intermediate vs. low *)	**0.62 (0.41–0.94)**	**0.027**	1.79 (0.84–3.79)	0.127
Monthly income (high vs. low *)	**0.36 (0.22–0.60)**	**<0.001**	0.62 (0.23–1.70)	0.361
The model entered the following variables: age, BMI, dyslipidemia, education level, family history of CVD, gender, hypertension, income, lipid-lowering medications, LMDS, marital status, smoking status, and work status.				

* Reference group. Abbreviations: BMI: body mass index; CI: confidence interval; CVD: cardiovascular disease; LMDS: Lebanese Mediterranean Diet Score; ORa: adjusted odds ratio; SD: standard deviation. ***p*-values in bold:** Statistically significant values.

**Table 4:** Multivariable analysis stratified by having hyperlipidemia.

Logistic Regression Taking CVD as the Dependent Variable				
	Not Having Hyperlipidemia		Having Hyperlipidemia	
	ORa (95% CI)	*p*-Value	ORa (95% CI)	*p*-Value
Gender (female vs. male *)	1.02 (0.72–1.44)	0.880	0.52 (0.29–0.93)	0.030
Marital status (married vs. single *)	0.82 (0.58–1.17)	0.286	0.83 (0.44–1.55)	0.571
Education level (university level vs. school level *)	1.14 (0.77–1.69)	0.504	0.94 (0.50–1.75)	0.846
Work status (employed vs. unemployed *)	0.76 (0.53–1.10)	0.152	0.80 (0.41–1.56)	0.530
Age	0.99 (0.98–1.00)	0.406	0.99 (0.97–1.01)	0.653
LMDS	**1.07 (1.04–1.11)**	**<0.001**	1.02 (0.96–1.07)	0.483
BMI	1.01 (0.98–1.05)	0.259	0.98 (0.93–1.04)	0.602
Hypertension (yes vs. no *)	**1.79 (1.22–2.62)**	**0.003**	1.69 (0.93–3.04)	0.081
Diabetes (yes vs. no *)	**2.14 (1.31–3.51)**	**0.002**	1.27 (0.65–2.47)	0.469
Family history of CVD (yes vs. no *)	**1.48 (1.07–2.04)**	**0.016**	**1.81 (1.08–3.02)**	**0.023**
Smoking status (previous vs. no *)	**1.95 (1.36–2.80)**	**<0.001**	1.06 (0.60–1.88)	0.821
Smoking status (yes vs. no *)	**1.98 (1.09–3.59)**	**0.024**	**2.29 (1.03–5.07)**	**0.041**
Hypoglycemic medications (yes vs. no *)	0.81 (0.39–1.71)	0.596	1.45 (0.66–3.17)	0.349
Monthly income (lower intermediate vs. low *)	0.73 (0.48–1.12)	0.155	**0.40 (0.20–0.79)**	**0.008**
Monthly income (higher intermediate vs. low *)	0.92 (0.60–1.41)	0.730	0.56 (0.28–1.13)	0.107
Monthly income (high vs. low *)	**0.39 (0.22–0.67)**	**0.001**	**0.35 (0.15–0.81)**	**0.014**
The model entered the following variables: age, BMI, diabetes, education level, family history of CVD, gender, hypoglycemic and hypertension medications, income, LMDS, marital status, smoking status, and work status.				

* Reference group. Abbreviations: BMI: body mass index; CI: confidence interval; CVD: cardiovascular disease; LMDS: Lebanese Mediterranean Diet Score; ORa: adjusted odds ratio. ***p*-values in bold:** Statistically significant values.

## Data Availability

The datasets used and/or analyzed during the current study are available from the corresponding author upon reasonable request due to ongoing research and planned follow-up studies.

## List of Abbreviations

ASCVD	Atherosclerotic cardiovascular diseases
BDS-22	Beirut Distress Scale
BMI	Body mass index
CV	Cardiovascular
CVD	Cardiovascular diseases
DM	Diabetes mellitus
DPP4	Dipeptidylpeptidase-4 inhibitors
GLP-1 RA	Glucagon-like peptide-1 receptor agonists
HbA1c	Hemoglobin A1c
HF	Heart failure
LDL	Low-density lipoprotein
LLT	Lipid-lowering therapy
LMDS	Lebanese Mediterranean Diet Score
MedDiet	Mediterranean diet
MI	Myocardial infarction
ORa	Adjusted odds ratio
RCBG	Random capillary blood glucose
RCT	Randomized controlled trials
SD	Standard deviation
SGLT2	Sodium-glucose cotransporter-2 inhibitors
SES	Socioeconomic status
TG	Triglyceride
T2DM	Type 2 diabetes mellitus
US	United States
WC	Waist circumference
